# Trends in Expanded-Spectrum Cephalosporin-Resistant *Escherichia coli* and *Klebsiella pneumoniae* among Dutch Clinical Isolates, from 2008 to 2012

**DOI:** 10.1371/journal.pone.0138088

**Published:** 2015-09-18

**Authors:** Matthijs van der Steen, Tjalling Leenstra, Jan A. J. W. Kluytmans, Akke K. van der Bij, ISIS-AR study group

**Affiliations:** 1 Centre for Infectious disease control (CIb), National Institute for Public Health and the Environment (RIVM), Bilthoven, the Netherlands; 2 Faculty of Earth and Life Sciences, VU University, Amsterdam, the Netherlands; 3 Department of Medical Microbiology and Infection Control, VU Medical Center (VUmc), Amsterdam, the Netherlands; 4 Laboratory for Microbiology and Infection Control, Amphia Hospital, Breda, The Netherlands; 5 Department of Medical Microbiology, Reinier de Graaf Groep, Delft, the Netherlands; Animal Health and Veterinary Laboratories Agency, UNITED KINGDOM

## Abstract

We investigated time trends in extended-spectrum cephalosporin-resistant *Escherichia coli* and *Klebsiella pneumoniae* isolates from different patient settings in The Netherlands from 2008–2012. *E*. *coli* and *K*. *pneumoniae* isolates from blood and urine samples of patients > = 18 years were selected from the Dutch Infectious Disease Surveillance System-Antimicrobial Resistance (ISIS-AR) database. We used multivariable Poisson regression to study the rate per year of blood stream infections by susceptible and resistant isolates, and generalized estimating equation (GEE) log-binomial regression for trends in the proportion of extended-spectrum cephalosporin-resistant isolates. Susceptibility data of 197,513 *E*. *coli* and 38,244 *K*. *pneumoniae* isolates were included. The proportion of extended-spectrum cephalosporin-resistant *E*. *coli* and *K*. *pneumoniae* isolates from urine and blood samples increased in all patient settings, except for *K*. *pneumoniae* isolates from patients admitted to intensive care units. For *K*. *pneumoniae*, there was a different time trend between various patient groups (p<0.01), with a significantly higher increase in extended-spectrum cephalosporin-resistant isolates from patients attending a general practitioner than in isolates from hospitalized patients. For *E*. *coli*, the increasing time trends did not differ among different patient groups. This nationwide study shows a general increase in extended-spectrum cephalosporin-resistant *E*. *coli* and *K*. *pneumoniae* isolates. However, differences in trends between *E*. *coli* en *K*. *pneumoniae* underline the importance of *E*. *coli* as a community-pathogen and its subsequent influence on hospital resistance level, while for *K*. *pneumoniae* the level of resistance within the hospital seems less influenced by the resistance trends in the community.

## Introduction

Treatment of infections by *Escherichia coli* and *Klebsiella pneumoniae* has become more challenging because of increasing resistance to extended-spectrum cephalosporin resulting from the production of Extended-Spectrum β-lactamases (ESBLs) [1]. Resistance has important implications for clinicians and patients due to a higher chance of inadequate treatment, increased length of hospital stay and additional healthcare costs [2,3,4]. Traditionally, long-term hospitalization, admission on an intensive care unit (ICU) and the presence of invasive medical devices were considered risk factors for colonization or infection with ESBL-producing organisms [1]. Since CTX-M ESBL types have replaced TEM and SHV mutants as the predominant ESBL type in Europe [5], the epidemiology of infections by ESBL-producing bacteria has changed from predominantly healthcare-associated risk factors to community-associated risk factors [6], and from *K*. *pneumoniae* to *E*. *coli* as the main hosting pathogen [1,5,6]. ESBL-producing *E*. *coli* isolates are increasingly detected in nursing homes and community-based health-care facilities and have been associated with travel [7]. This facilitates a potential influx of ESBL-producing microorganisms from the community into the hospital [6,8].

This worldwide spread of ESBL-producers into the community [9,10] possibly requires new approaches for the surveillance and prevention of ESBL-producing *E*. *coli* and *K*. *pneumoniae* in hospital settings.

We therefore assessed trends in extended-spectrum cephalosporin-resistant *K*. *pneumoniae*, representing nosocomial resistance and *E*. *coli* isolates, representing community-onset resistance, from hospitalized patients as well as patients attending a general practitioner (GP).

## Materials and Methods

### Data

We used data from the Dutch Infectious Disease Surveillance Information System–Antibiotic Resistance (ISIS-AR) over a five-year period (2008 to 2012). The ISIS-AR database is described in detail elsewhere [11]. Briefly, the 30 currently participating laboratories cover approximately 65% of the Dutch laboratories with a wide geographic range and provide on a monthly basis data on bacterial identification and antimicrobial susceptibility of all routinely cultured isolates. Over 50% of the Dutch population is covered by ISIS-AR and its antimicrobial susceptibility data are considered representative for the Netherlands. The data include Minimum Inhibitory Concentrations (MICs) of automated susceptibility testing systems or E-tests and patient data (i.e., age, gender, hospital department, specimen site). Data quality is assured by structural quality control and monthly feedback of unusual findings. Additionally laboratories are regularly audited by independent external experts of the Dutch institute for the promotion of quality in laboratory research and for the accreditation of laboratories in the health care sector (CCKL [http://www.cckl.nl/]).

### Isolates

*E*. *coli* and *K*. *pneumoniae* isolates from blood and urine samples of adult patients (i.e., age > = 18 years) attending a general practitioner (GP) or an outpatient department (OPD) or admitted at an inpatient department (IPD) or intensive care unit (ICU) were included. For some hospitals included in the ISIS-AR database no distinction between the emergency department and OPD/IPD can be made, resulting in isolates from emergency departments to be included in both OPD and IPD settings. Only isolates from continuously reporting laboratories over the period 2008 to 2012 were selected, resulting in the inclusion of data from 22 participating laboratories, still representing over 40% of the Dutch population). MIC results of automated susceptibility testing systems for cefotaxime/ceftriaxone or ceftazidime were re-interpret using the European Committee for Antimicrobial Susceptibility Testing (EUCAST) 2012 breakpoints (version 2.0 valid until Dec 31, 2012) for classifying organisms as susceptible or resistant, including non-susceptible isolates [12]. Isolates were defined as extended-spectrum cephalosporin resistant if they were non-susceptible to at least one of the two types of agents recommended for ESBL screening in The Netherlands, namely cefotaxime/ceftriaxone and ceftazidime. To avoid overrepresentation caused by multiple isolates from the same patient, isolates obtained in the 30-day period following the first positive blood isolate were excluded and we assumed that each blood isolate included represented a bacteremia episode. For urine isolates, the first isolate per patient per year was included, with at least a 90 days period between the included isolate and an isolate selected from the previous year [13].

### Statistical analyses

First, we calculated the time trend in the rate of bloodstream infections by *E*. *coli* and *K*. *pneumoniae* per year for extended-spectrum cephalosporin susceptible (ESC-S) and resistant isolates combined and for resistant isolates separately. The rate was defined as the absolute number of isolates, assuming a stable at-risk population. The yearly rate ratio (yRaR) was calculated using multivariable Poisson regression, forcing a log-linear trend over time, with a robust standard error and adjusted for patient characteristics (i.e., age, gender, patient setting and the geographic location of the laboratory within the Netherlands, see Table 1 for definitions). Secondly, we analyzed the time trend in the yearly proportion of extended-spectrum cephalosporin resistant *E*. *coli* and *K*. *pneumoniae* isolates (expressed as yearly risk ratios [yRiR) by a multivariable log-binomial regression analysis, that included patient characteristics. These analyses were performed separately for blood samples, urine samples of female patients and urine samples of male patients. Urine isolates from male and female patients were analyzed separately, because the Dutch College of General Practitioners guidelines on urinary tract infections recommend routine culture of urine for all males with urinary tract symptoms, since urinary tract infections in males are considered complicated infections. For females, testing is only recommend in case of non-response to first-line antimicrobial treatment or in case of a complicated infection [14].

**Table 1:** Background characteristics for *Escherichia coli* and *Klebsiella pneumoniae* isolates, ISIS-AR The Netherlands, 2008–2012.

	*E*. *coli*						*K*. *pneumoniae*					
	No. (%)		No. (%)		No. (%)		No. (%)		No. (%)		No. (%)	
	blood isolates		male urine isolates		female urine isolates		blood isolates		male urine isolates		female urine isolates	
Variable	*n* = 14461		*n* = 42270		*n* = 140782		*n* = 2492		*n* = 9995		*n* = 25553	
ESC-R^a^	863	(6.0)	2481	(5.2)	5331	(3.6)	204	(8.2)	631	(6.3)	1000	(3.8)
Age in years												
19–64	4049	(28.0)	19379	(41.0)	69121	(47.3)	774	(31.1)	3169	(31.7)	8902	(33.5)
≥65	10412	(72.0)	27891	(59.0)	76992	(52.7)	1718	(68.9)	6826	(68.3)	17651	(66.5)
Gender^b^												
Female	7282	(50.4)	-	-	-	-	1087	(43.6)	-	-	-	-
Male	7179	(49.6)	-	-	-	-	1405	(56.4)	-	-	-	-
Patient setting^c^												
GP	-	-	24244	(51.3)	89704	(61.4)	-	-	3702	(37.0)	14494	(54.6)
OPD	4630	(32.0)	13045	(27.6)	29640	(20.3)	690	(27.7)	3728	(37.3)	6737	(25.4)
IPD	8638	(59.7)	9558	(20.2)	25885	(17.7)	1515	(60.8)	2419	(24.2)	5042	(19.0)
ICU	1186	(8.2)	423	(0.9)	884	(0.6)	287	(11.5)	146	(1.5)	280	(1.1)
Geographic Region												
Northwest	3727	(25.8)	15384	(32.5)	47351	(32.4)	618	(24.8)	2604	(26.1)	6440	(24.3)
Northeast	1953	(13.5)	2200	(4.7)	5346	(3.7)	259	(10.4)	1420	(14.2)	4295	(16.2)
Middle	2523	(17.4)	9991	(21.2)	31233	(21.4)	388	(15.6)	1559	(15.6)	4119	(15.5)
Southwest	1814	(12.5)	7860	(16.7)	26901	(18.4)	347	(13.9)	1165	(11.7)	3316	(12.5)
South	4423	(30.6)	11766	(24.9)	35018	(24.0)	873	(35.0)	3233	(32.3)	8358	(31.5)

^a^ESC-R: Extended-spectrum cephalosporin-resistant

^b^Urine samples are stratified for gender.

^c^The patient settings are: General Practitioner (GP), Outpatient Department (OPD), Inpatient Department (IPD) and Intensive Care Unit (ICU). The dataset did not contain blood isolates from general practitioners.

To determine whether time trends between the different patient settings (i.e., GP, OPD, IPD and ICU) differed significantly, an interaction term between patient setting and time in years was tested. To correct for repeated measures of patients contributing more than once to the dataset, generalized estimating equation (GEE) was used. This GEE analyses was used and for all patient categories and settings, except for the analysis of urine isolates from female patients admitted at the ICU departments, due to the low number of repeated measures. A limit of six repeated measures was set to avoid sparse data problems, which implied the exclusion of less than 1% of all isolates. Proportions point-estimates with 95% confidence intervals and time trends were plotted using time in years as a categorical variable. A p-value <0.05% was considered as statistically significant. Data analysis was performed using SPSS software version 19 (IBM) and Stata/SE software version 12 (StataCorp).

### Ethics statement

The data of the bacterial isolates and their susceptibility results used in this study belong to the microbiological laboratories participating in ISIS-AR and was obtained as part of routine clinical care in the past. Written or verbal consent of patients was therefore not obtained. Furthermore, all patient identifiers had been previously removed and data were analysed anonymously. According to the Dutch Medical Research Involving Human Subjects Act (WMO) this study was considered exempt from review by an Institutional Review Board.

## Results

### Characteristics

Table 1 summarizes the background characteristics of isolates included in this study. The number of included *E*. *coli* isolates (*n* = 197,513) was substantially higher than the number of included *K*. *pneumoniae* isolates (*n* = 38,040). Most of the isolates originated from urine samples of female patients. The overall proportion of extended-spectrum cephalosporin resistant isolates was lower in *E*. *coli* isolates (6.0, 5.2 and 3.6%, for blood, urine from males and urine from females respectively) compared to *K*. *pneumoniae* isolates (8.2, 6.3 and 3.8%, respectively). Resistance was highest among blood isolates and lowest among urine isolates of female patients.

### Time trends in the rate of bloodstream infections

**Fig 1:**
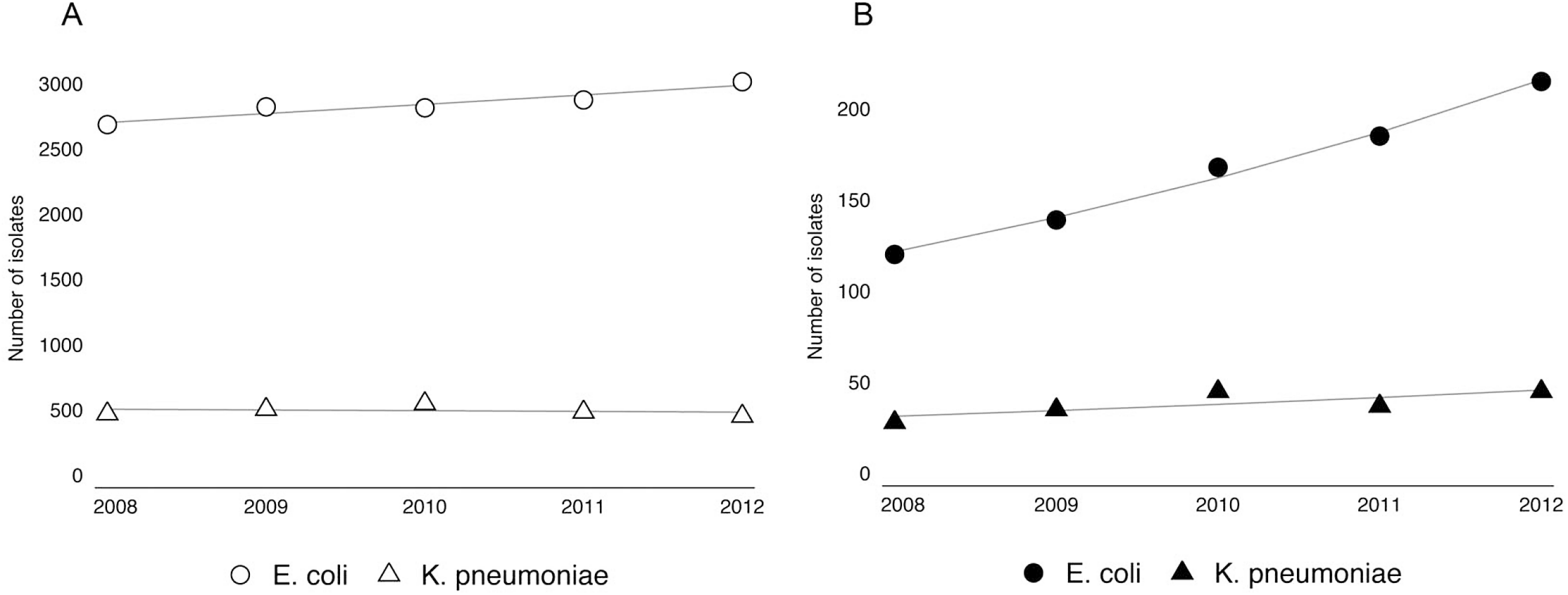
The rate of bloodstream infections by *Escherichia coli* and *Klebsiella pneumoniae* (A) and the rate of bloodstream infections by extended-spectrum cephalosporin-resistant *Escherichia coli* and *Klebsiella pneumoniae* (B) from 2008–2012, ISIS-AR, the Netherlands, adjusted for patient characteristics, including age, gender, patient setting and geographic region. The plots show the point-estimates and log-linear trends generated by a Poisson regression with robust standard error.

Fig 1 shows a significant increase in the total number of *E*. *coli* bloodstream infections per year from 2008–2012; from 2687 in 2008 to 3016 in 2012 (yRaR 1.03 [95% confidence interval (95% CI) 1.03–1.03]), while there was no time trend in the total number of bloodstream infections by *K*. *pneumoniae*, from 472 in 2008 to 454 in 2012 (yRaR 0.99 [95% CI 0.95–1.03]). When limiting analysis to extended-spectrum cephalosporin resistant isolates, there was an increase in the total number of bloodstream infections by both *E*. *coli* (yRaR 1.15 [95% CI 1.09–1.22]) and *K*. *pneumoniae* (yRaR 1.09 [95% CI 1.09–1.10]). The degree of increase in resistant *E*. *coli* isolates was higher than the increase in susceptible isolates (Fig 1).

### Trends in the proportion of extended-spectrum cephalosporin resistance

**Fig 2:**
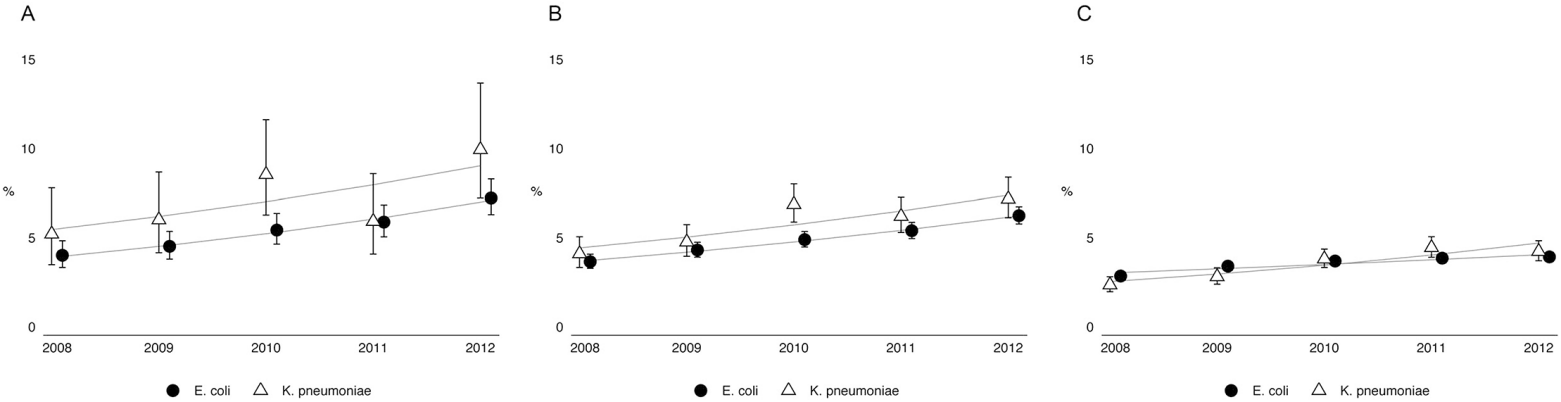
The proportion (in %) of extended-spectrum cephalosporin-resistant *Escherichia coli* and *Klebsiella pneumoniae* isolates, for blood isolates (A), male urine isolates (B) and female urine isolates (C) from 2008–2012, ISIS-AR, the Netherlands. The point-estimates and log-linear trends per year were estimated with a generalized estimation equation (GEE) for log-binominal regression including time in years, and patient characteristics, including age, gender, patient setting and geographic region. The error bars represent the 95% confident intervals of each of the point-estimates.

There was a significant increase in the proportion of extended-spectrum cephalosporin resistant *E*. *coli* and *K*. *pneumoniae* isolates (Table 2 and Fig 2). The yearly increase in the proportion of resistant *E*. *coli* in blood isolates (yRiR 1.15 [95% CI 1.09–1.20]) and *K*. *pneumoniae* in blood isolates (yRiR 1.11 [95% CI 1.00–1.23]) was similar. The increase in resistance among *E*. *coli* and *K*. *pneumoniae* was also similar for urine isolates from male patients (Table 2). However, when analyzing urine isolates from female patients there was a stronger increase in resistant *K*. *pneumoniae* isolates than in resistant *E*. *coli* isolates.

**Table 2:** Adjusted risk ratio’s (yRiR) per year for the time trends in proportion of *Escherichia coli* and *Klebsiella pneumoniae* isolates resistant to third generation cephalosporins, ISIS-AR, The Netherlands, 2008–2012.

	yRiR^a^	[95% CI]
**Blood**		
*E*. *coli*	1.15	[1.09–1.20]
*K*. *pneumoniae*	1.11	[1.00–1.23]
**Urine from males**		
*E*. *coli*	1.13	[1.10–1.16]
*K*. *pneumoniae*	1.12	[1.07–1.18]
**Urine from females**		
*E*. *coli*	1.07	[1.05–1.09]
*K*. *pneumoniae*	1.16	[1.11–1.21]

^a^Adjusted yearly risk ratio’s (yRiR) and 95% confidence intervals (95% CI) are generated with a log-binomial regression, adjusted for patient characteristics, including age, gender, patient setting and geographic region, and corrected for repeated measures using generalized estimating equation (GEE).

### Trends in the proportion of extended-spectrum cephalosporin resistant isolates per patient setting

**Fig 3:**
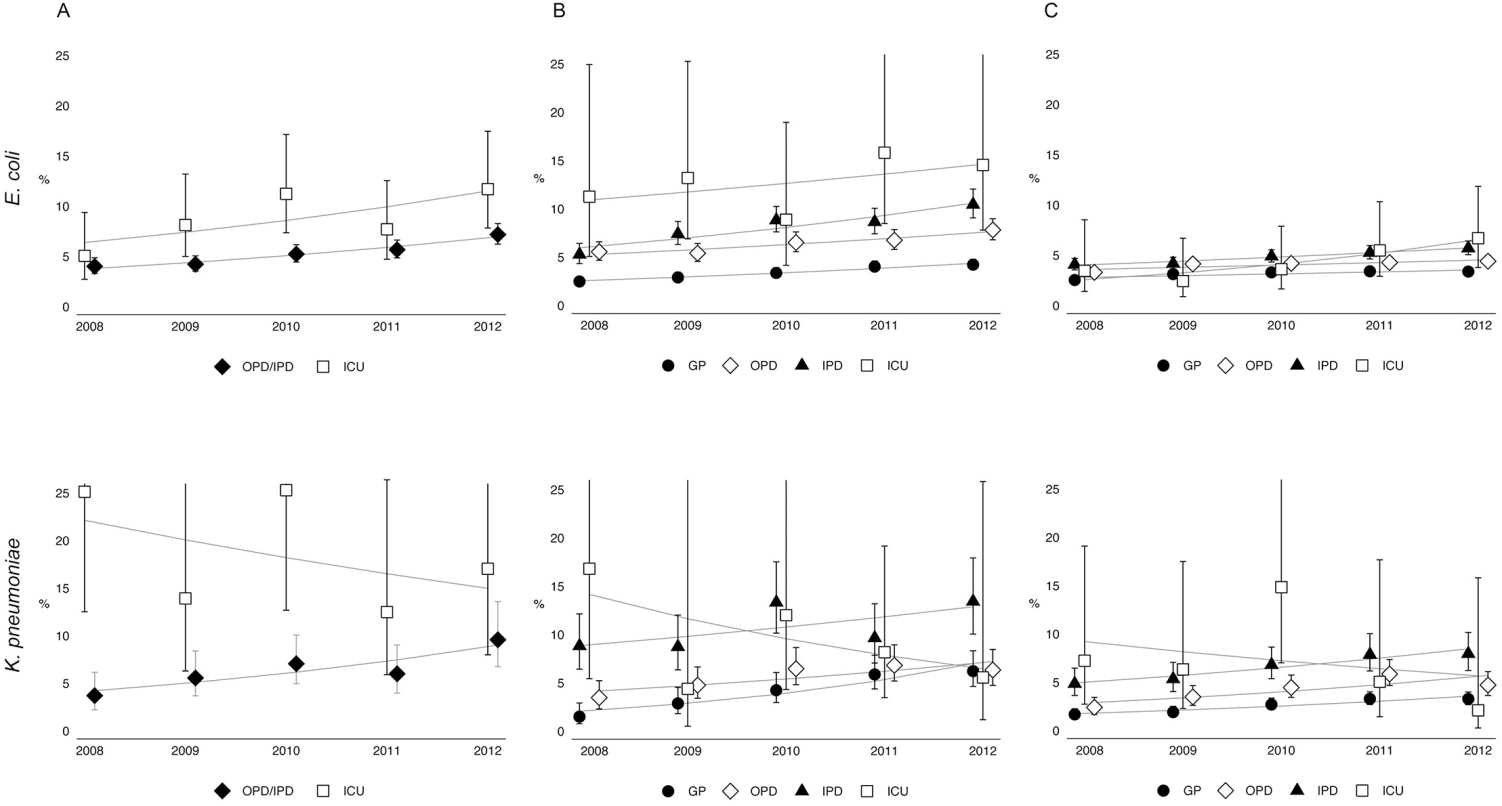
The proportion (in %) extended-spectrum cephalosporin-resistant *Escherichia coli* and *Klebsiella pneumoniae* isolates, for blood isolates (A), male urine isolates (B) and female urine isolates (C), per patient setting (general practitioner [GP], outpatient department [OPD], inpatient department [IPD] and intensive care units [ICU]), from 2008–2012, ISIS-AR, the Netherlands. The point-estimates are generated with a generalized estimation equation (GEE) for log-binominal regression, including time in years, and patient characteristics, including age, gender, and geographic region. The error bars represent the 95% confident intervals of each of the point-estimates. To produce plots with comparable y-axis, the upper error bar is cut-off for some of the point-estimates.

Fig 3 and Table 3 show the point-estimates and log-linear trends in the proportion of extended-spectrum cephalosporin resistant isolates per patient setting, namely GP, OPD, IPD and ICU. There was a general tendency towards increasing resistance for both *E*. *coli* and *K*. *pneumoniae* isolates from all patient settings, except for *K*. *pneumoniae* isolates of patients admitted at an ICU. Furthermore, within the different sample types, there were differences in time trends between both organisms when analyzing per setting. For example, the increase in the proportion of extended-spectrum cephalosporin resistant *E*. *coli* isolates from male urine samples was present in all patient settings and did not differ significantly between the different settings (p = 0.77). However, for *K*. *pneumoniae*, there was a significant difference in time trend in the proportion of resistance between isolates from hospital sites and isolates from the GP (p<0.01 [Table 3]); the increase in resistance was significantly higher among isolates from the GP. For isolates from females, a similar pattern was seen, although not statistically significant. For blood isolates, there was a difference in the resistance time trend between *K*. *pneumoniae* isolates from outpatient and hospital departments and isolates from the ICUs (p = 0.07 [Table 3]), while there were no differences in time trend between the different hospital settings for *E*. *coli* isolates (p = 0.84).

**Table 3:** Adjusted risk ratio’s (yRiR) per year for the time trends in proportion of *Escherichia coli* and *Klebsiella pneumoniae isolates* resistant to third generation cephalosporins per patient setting, ISIS-AR, The Netherlands, 2008–2012.

	*E*. *coli*		*K*. *pneumoniae*	
	yRiR^a^	[95% CI]	yRiR	^a^ [95% CI]
**Blood**				
OPD/IPD^b^	1.15	[1.10–1.21]	1.19	[1.06–1.35]
ICU	1.14	[1.00–1.31]	0.92	[0.77–1.10]
**Urine from males**				
GP	1.14	[1.08–1.19]	1.34	[1.18–1.51]
OPD	1.09	[1.04–1.14]	1.14	[1.03–1.23]
IPD	1.14	[1.09–1.19]	1.08	[1.00–1.18]
ICU	1.06	[0.88–1.29]	0.85	[0.61–1.17]
**Urine from females**				
GP	1.06	[1.03–1.09]	1.19	[1.10–1.28]
OPD	1.06	[1.02–1.10]	1.17	[1.08–1.28]
IPD	1.09	[1.05–1.13]	1.13	[1.05–1.22]
ICU	1.25	[0.99–1.57]	0.91	[0.86–1.21]

^a^Adjusted risk ratio’s (yRiR) and 95% confidence intervals (95% CI) are generated with a log-binomial regression, adjusted patient characteristics, including age, gender and geographic region, and corrected for repeated measures using generalized estimating equation (GEE).

The patient settings are: General Practitioner (GP), Outpatient Department (OPD), Inpatient Department (IPD) and Intensive Care Unit (ICU)

^b^ Because the absence of blood samples from GPs and the low number of isolates from OPD and IPD this both hospital locations were combined.

## Discussion

Recent literature suggests a shift in the source of ESBL-producing bacteria from mainly hospital-acquired towards the emergence of these microorganisms in the community [1,6,15]. This study focused on differences in time trends between extended-spectrum cephalosporin resistant *E*. *coli* and *K*. *pneumoniae* isolates from different patient settings. First, time trends in the rate of bloodstream infections were studied, showing no increase in the total number of *K*. *pneumoniae* infections, while the number of infections with *E*. *coli* increased. The rate of bloodstream infections by extended-spectrum cephalosporin resistant *E*. *coli* and *K*. *pneumoniae* increased for both species. The proportion of extended-spectrum cephalosporin-resistant *E*. *coli* isolates from urine and blood samples increased in all patient settings in contrast to *K*. *pneumoniae* isolates showing different time trends between various patient settings. For *K*. *pneumoniae* isolates there was a significantly higher increase in resistant isolates from patients attending a general practitioner than in isolates from hospitalized patients. Especially in the ICU departments, there was a clear difference in time trend between both species, with an increase in the proportion of resistant *E*. *coli* isolates and a non-significant decrease in resistance in *K*. *pneumoniae* isolates.

The lower rise in the proportion of extended-spectrum cephalosporin resistant *K*. *pneumoniae* isolates in hospital setting indicates that perhaps, a plateau has been reached. However, due to the small timespan and the low numbers of *K*. *pneumoniae* isolates in this study, it might also reflect a temporal decline in the percentage of resistant isolates as shown in a recent study by Kronenberg *et al*., [16]. They showed that the increasing resistance time trends for both *E*. *coli* and *K*. *pneumoniae* are similar in Switzerland from 2004–2011. However, the percentages of resistant *K*. *pneumoniae* isolates showed more variations then the yearly percentages of resistant *E*. *coli*, due to multiple outbreaks in several institutions [16]. On the other hand, a recent study from the United States, studying *K*. *pneumoniae* isolates from 1999 to 2010, showed a plateauing trend of extended-spectrum cephalosporin resistant *K*. *pneumoniae* isolates [13]. Additionally, data from resistance surveillance of bloodstream infections in the United Kingdom (UK), 1990–2010, describes a dip in ESBL prevalence among *E*. *coli* isolates around 2006 with a stable epidemiology thereafter [17]. The author suggests that this effect might reflect the massive prescribing changes in UK hospitals, with a move away from cephalosporins and quinolones towards beta-lactamase inhibitor combinations. These prescribing changes, like reducing the number of Methicillin-Resistant *Staphylococcus aureus* (MRSA), became a major subject in national reduction targets [17] and the simultaneous increased attention on contact precautions for patients infected with or carrying MRSA, might also have benefitted the prevention of spread of resistant *E*. *coli* and *K*. *pneumoniae* isolates.

The Dutch Working Party on Antibiotic Policy (SWAB) and the Dutch Working Party on Infection Prevention (WIP) fight the same battle for rational and restrained antibiotic policy and the control of highly resistant microorganisms (HRMO) in Dutch hospitals as the UK [18,19]. Data from the European Antimicrobial Surveillance Network (EARS-net) shows a steady, almost linear, rise in the percentages of extended-spectrum cephalosporin resistance in *E*. *coli* isolates between neighboring European countries, including the Netherlands and the UK [20]. However, a more variable pattern is visible for *K*. *pneumoniae* isolates and the lowest percentages of resistance were found in the UK and the Netherlands in 2012, which might reflect the common efforts in infection control.

Differences in time trends between *E*. *coli* and *K*. *pneumoniae* isolates likely reflect the different characteristics of these two bacteria. *K*. *pneumoniae* is a bacterium that has adapted especially to the hospital environment and survives longer than other Enterobacteriaceae on hands and environmental surfaces, making it easier to cause cross-infections within hospitals [21, 22]. *E*. *coli* is ubiquitous in the community and therefore less affected by infection prevention policies. Subsequently, the rise in extended-spectrum cephalosporin resistance within hospital settings is probably explained by the rise of resistant *E*. *coli* isolates within the community. In elderly patients from long-term care facilities in the United States, sequence type (ST) 131, a clone of extended-spectrum cephalosporin resistant *E*. *coli* that has been implicated as an important source of the emergence of extended-spectrum cephalosporin resistance in *E*. *coli*, has become dominant [23, 24]. Additionally, it has been suggested that the ST131 clone has become more common in the community due to the antibiotic pressure [7, 24, 25].

This study has some limitations, including the limited timespan of the study and the low number of resistant isolates, in particular for *K*. *pneumoniae*, due to low prevalence of resistance in the Netherlands. Results found might therefore not be representative for countries with higher levels of resistance. Furthermore, sampling bias might have resulted in the overrepresentation of resistant isolates. However, this bias is likely to have been constant over time, resulting in no effect on the yearly trend. Third, the data has been corrected for repeated measures. Since there is no standardized patient identification across laboratories within our dataset, unidentified double sampling of individual persons may have occurred. However, we assume that the effects of double sampling are limited considering the organization of healthcare in The Netherlands, where patients generally have one GP. Fourth, for comparing differences in time trends between community-onset and hospital-associated infections and resistant bacteria it would be preferable to have better coverage of patients within long-term care facilities, given their potential role in spread of resistance [23]. Fifth, to confirm that the influx of ESBL-producing bacteria from the community into hospital settings occurs, genotypic information is needed, whereas ISIS-AR only includes phenotypic data. On the other hand, results in our study are robust due to the large numbers of isolates present in the dataset and the wide geographic distribution of the participating laboratories ensuring a good representation of the Dutch population. By the inclusion of laboratories that have continuously delivered data it was possible to compare the year-to-year proportion and to give reliable time trends.

## Conclusion

This nationwide study shows a general increase in extended-spectrum cephalosporin-resistant *E*. *coli* and *K*. *pneumoniae* isolates. However, differences in trends between *E*. *coli* en *K*. *pneumoniae* underline the importance of *E*. *coli* as a community-pathogen and its subsequent influence on hospital resistance level, while for *K*. *pneumoniae* the level of resistance within the hospital seems less influenced by the resistance trends in the community.

This increase in the proportion of resistant *E*. *coli* isolates within the community and hospital settings implies that preventing the spread of extended-spectrum cephalosporin-resistance should not only focus on hospital settings, but also on preventing the spread of resistant species within the community. These efforts could prevent the influx of resistant species into the hospital setting and help to reduce the chance of inadequate treatment, increased length of hospital stay and additional healthcare costs due to infection with extended-spectrum cephalosporin-resistance bacteria.
